# Serum and cerebrospinal fluid neurofilament light chain in patients with central nervous system infections caused by varicella-zoster virus

**DOI:** 10.1007/s13365-020-00889-2

**Published:** 2020-08-20

**Authors:** Tobias Tyrberg, Staffan Nilsson, Kaj Blennow, Henrik Zetterberg, Anna Grahn

**Affiliations:** 1 Department of Infectious Diseases, Institute of Biomedicine, The Sahlgrenska Academy at University of Gothenburg, Gothenburg, Sweden; 2 Department of Mathematical Statistics, Chalmers University of Technology, Gothenburg, Sweden; 3 Department of Psychiatry and Neurochemistry, Institute of Neuroscience and Physiology, The Sahlgrenska Academy at University of Gothenburg, Mölndal, Sweden; 4 Clinical Neurochemistry Laboratory, Sahlgrenska University Hospital, Mölndal, Sweden; 5 Department of Neurodegenerative Disease, UCL Institute of Neurology, Queen Square, London, UK; 6 UK Dementia Research Institute, London, UK

**Keywords:** Varicella-zoster virus (VZV), Central nervous system (CNS), Biomarker, Encephalitis

## Abstract

Varicella-zoster virus (VZV) is a common cause of viral central nervous system (CNS) infection, and patients may suffer from severe neurological sequelae. The biomarker neurofilament light chain (NFL) is used for assessment of neuronal damage and is normally measured in cerebrospinal fluid (CSF). Novel methods have given the possibility to measure NFL in serum instead, which could be a convenient tool to estimate severity of disease and prognosis in VZV CNS infections. Here, we investigate the correlation of serum and CSF NFL in patients with VZV CNS infection and the association of NFL levels in serum and CSF with different VZV CNS entities. NFL in serum and CSF was measured in 61 patients who were retrospectively identified with neurological symptoms and VZV DNA in CSF detected by PCR. Thirty-three herpes zoster patients and 40 healthy blood donors served as control groups. NFL levels in serum and CSF correlated strongly in the patients with VZV CNS infection. Encephalitis was associated with significantly higher levels of NFL in both serum and CSF compared with meningitis and Ramsay Hunt syndrome. Surprisingly, herpes zoster controls had very high serum NFL levels, comparable with those shown in encephalitis patients. We show that analysis of serum NFL can be used instead of CSF NFL for estimation of neuronal injury in patients with VZV CNS infection. However, high levels of serum NFL also in patients with herpes zoster, without signs of CNS involvement, may complicate the interpretation.

## Introduction

Varicella-zoster virus (VZV) is a neurotropic virus in the herpes family, and it is one of the most common viral agents causing infection in the central nervous system (CNS) (Granerod et al. 2010; Mailles et al. 2012; Persson et al. 2009). After primary infection, the virus establishes latency in sensory and autonomic ganglia. Reactivation can cause several different clinical manifestations involving the CNS, such as encephalitis, meningitis, myelitis, vasculitis, and cranial nerve affection, including Ramsay Hunt syndrome with peripheral facial palsy (Persson et al. 2009). Encephalitis is the most serious manifestation and is associated with both high mortality and morbidity (Mailles et al. 2012), but all VZV CNS manifestations may include serious neurological sequels (Persson et al. 2009). Diagnostic and prognostic tools are of importance to try to estimate the degree of CNS injury caused by the infection, and possibly also the need of rehabilitation. Different biomarkers measured in cerebrospinal fluid (CSF) or serum may be used for this purpose.

In patients with VZV CNS infection, viral load and some other biomarkers have been investigated, such as neurofilament light chain (NFL), glial fibrillary acidic protein and S-100B protein (Aberle et al. 2005; Grahn et al. 2013; Lindstrom et al. 2016; Persson et al. 2009). Viral load of VZV in CSF was in one study shown to be higher in encephalitis patients (Aberle et al. 2005), but could in another study not differ against meningitis patients and was not related to the outcome (Persson et al. 2009). NFL is a protein exclusively expressed in neurons and is used as a biomarker for neuroaxonal damage (Gaetani et al. 2019). Increased concentrations of CSF NFL are seen in patients with a wide range of neurodegenerative diseases (Lycke et al. 1998; Nylen et al. 2006; Nylen et al. 2002). CSF NFL concentrations have been reported to be higher in patients with VZV CNS infection compared with that of controls. This increase is most pronounced in patients with encephalitis, but is also seen in those with meningitis (Grahn et al. 2013) and Ramsay Hunt syndrome (Grahn et al. 2013; Lindstrom et al. 2016), and may be a reflection of the degree of CNS injury.

However, most biomarkers used in the diagnostics of CNS infections are based on analysis of CSF obtained through a lumbar puncture, a sometimes harmful procedure for the patient. During recent years, a new method for analysis of biomarkers in serum has been introduced. The novel ultra-sensitive Single-molecule array (Simoa) method, which is a digital enzyme-linked immunosorbent assay (ELISA) (Cohen and Walt 2017), has been shown to be a both reliable and compelling alternative for analysis of NFL in patients with CNS disease (Gisslen et al. 2016). A strong correlation has been found between concentrations of NFL in blood and CSF in neurodegenerative diseases (Disanto et al. 2017; Gaiottino et al. 2013) and HIV-related CNS injury (Gisslen et al. 2016). Yet, the usefulness of measuring serum NFL concentrations in patients with viral CNS infections is scarcely elucidated, and no studies have so far evaluated this method in patients with VZV CNS infection.

Our aim was to study the correlation between serum and CSF NFL concentrations in patients with VZV CNS infection, and to relate the NFL levels to clinical manifestations and parameters.

## Methods

### Patients and controls

CSF samples with VZV DNA-positive by PCR were identified between 2007 and 2014 at the Department of Virology at Sahlgrenska University Hospital in Gothenburg, Sweden. The samples had been drawn in the clinical setting due to CNS symptoms from patients admitted to the Sahlgrenska University Hospital. For comparison, paired CSF and serum samples were identified, i.e. serum taken within 2 days of CSF sample. Clinical information was obtained through medical records. Patients with detectable VZV DNA in CSF and symptoms suggestive of CNS involvement were considered to have a VZV CNS infection. The patients were categorised based on clinical symptoms and criteria published earlier (Grahn et al. 2013) into either encephalitis, meningitis or Ramsay Hunt syndrome. Patients who did not meet the criteria for any of these groups were categorised as “other neurological symptoms”. Exclusion criteria were lack of consent and other concomitant CNS disease.

Two control groups were defined. One group of 33 patients, who had been admitted for herpes zoster without CNS symptoms to Sahlgrenska University Hospital between 2000 and 2013, was identified through a diagnosis registry. The inclusion criteria were herpes zoster without CNS manifestations and at least one serum sample available for analysis. The serum samples were drawn a median 4 days (range − 11–53) in relation to onset of disease. Thirty-one out of the 33 patients were sampled within − 2–15 days in relation to onset of disease. The second control group consisted of 40 healthy blood donors.

The study was approved by the medical ethics committee at Gothenburg University, and informed consent was obtained for inclusion in the study.

### Management of serum and CSF samples

Samples of CSF drawn at the acute stage of disease were analysed for VZV DNA with an in-house TaqMan PCR for quantitative analysis of viral load (Persson et al. 2009). The CSF and serum samples were stored at − 20 °C. In 2017, 48 of the CSF samples were analysed for NFL using the NF-Light kit from UmanDiagnostics, according to instructions by the kit manufacturer (UmanDiagnostics, Umeå, Sweden). In 2018, 9 additional CSF samples (2 encephalitis, 2 meningitis, 4 Ramsay Hunt syndrome, 1 “other neurological symptoms”) were analysed for NFL using an in-house method (Gaetani et al. 2018). As previously described in detail (Gaetani et al. 2018), this method is highly correlated with the UmanDiagnostics assay (*r* = 0.9984, *p* < 0.001, no offset), which was verified in a Passing-Bablok analysis. Serum NFL concentration was measured using an in-house digital ELISA on the Simoa platform as previously described in detail (Gisslen et al. 2016). Albumin ratio was calculated as a measure of blood-brain barrier damage (CSF albumin (mg/L)/serum albumin (g/L)). When comparing CSF NFL levels between patient groups, we performed an age adjustment of CSF NFL based on the formula (CSF NFL (pg/mL) = 97.5 × 1.031^age^) (Yilmaz et al. 2017).

### Statistical analysis

Serum and CSF NFL concentrations were log-transformed prior to analysis to obtain a normal distribution. NFL concentrations are presented as median and interquartile range (IQR) or geometric mean and 95% confidence interval. Comparison of raw NFL concentrations in CSF and serum as well as age-adjusted CSF NFL (Yilmaz et al. 2017) was made using one-way ANOVA with Tukey’s multiple comparison test. ANCOVA was used to adjust for group differences in serum and CSF NFL correlation, and to adjust for age when comparing serum NFL levels. Pearson correlation analysis was used for correlations. Prism version 7.0d (GraphPad Software Inc., La Jolla, CA), SPSS version 25 (IBM, Armonk, NY) and R version 3.3.1 were used for statistical calculations.

## Results

### Patients and controls

Sixty-one consenting patients with detectable VZV DNA in CSF and neurological symptoms related to VZV infection were included in the study. Nine of the included patients were only analysed for either serum NFL or CSF NFL due to insufficient sample volumes (serum NFL only (*n* = 4), CSF NFL only (*n* = 5)). Both serum and CSF samples were drawn a median 4 days (range 0–90) after onset of disease. The patient who was sampled 90 days after onset of symptoms was a 38-year-old man who presented with slowly progressive bilateral proximal weakness in arms and legs for the last 3 months. After treatment with acyclovir and cortisone, the symptoms slowly regressed. Details about the study groups and comparison of clinical data are shown in Table 1. Notably, the patients with encephalitis in our material were significantly older than patients with meningitis and herpes zoster, and the healthy controls (*p* < 0.01 for all).

**Table 1:** Clinical data of patients with different manifestations of VZV CNS infection (*n* = 61) and controls (*n* = 73)

	Encephalitis	Meningitis	Ramsay Hunt syndrome	Other neurological symptoms	Herpes zoster controls	Healthy controls
*n*	17	28	9	7	33	40
Age, median (range)	73 (34–89)	32.5 (15–82)	57 (20–82)	47 (22–84)	41 (3–85)	54.5 (18–75)
Male/female	9/8	13/15	6/3	3/4	17/16	20/20
CSF VZV DNA, median (range)	22,000 (0–1.3 × 10^8^)^a^	21,600 (263–1.3 × 10^6^)	1300 (50–759,000)	1150 (100–11,000)	ND	ND
Albumin ratio, median (range)	24.2 (4.9–156.7)	19.2 (3.3–39.3)	11.3 (2.8–25)	12.1(5.6–19.3)	ND	ND
Immunocompromised, *n* (%)	4 (24)	5 (18)	0	2 (29)	17 (52)	ND
Rash, *n* (%)	7 (41)	12 (43)	7 (78)	5 (71)	33 (100)	ND

*CSF* cerebrospinal fluid, *VZV* varicella-zoster virus

^a^One sample with CSF VZV = 0 was drawn 25 days after onset of symptoms, and 21 days after the first CSF sample that was VZV DNA-positive by PCR

Seven patients who did not meet the criteria for any predefined group were categorised as “other neurological symptoms” which consisted of the following diagnoses: encephalopathy (*n* = 4), polyneuropathy (*n* = 1), radiculitis (*n* = 1) and cranial nerve ganglionitis (*n* = 1). Two patients with peripheral facial palsy that also reached criteria for meningitis were categorised as Ramsay Hunt syndrome. One patient with encephalitis presented with subarachnoid haemorrhage due to an aneurysm which was regarded as a VZV manifestation.

Eleven of the patients with VZV CNS infection were immunocompromised (vasculitis (*n* = 3), systemic lupus erythematosus (*n* = 2), leukaemia (*n* = 2), essential thrombocytosis (*n* = 1), lung malignancy (*n* = 1), rheumatoid arthritis (*n* = 1) and Crohn’s disease (*n* = 1)). Of the herpes zoster controls, 17 patients were immunocompromised (leukaemia (*n* = 6), lymphoma (*n* = 3), HIV (*n* = 3), rheumatoid arthritis (*n* = 1), Wilm’s tumour (*n* = 1), liver transplant (*n* = 1), kidney transplant (*n* = 1) and ulcerative colitis (*n* = 1)). All of these patients received immunosuppressing treatment except for six patients in the herpes zoster control group (HIV (*n* = 3), chronic lymphocytic leukaemia (*n* = 2), and lymphocytic lymphoma (*n* = 1)).

### NFL in serum and CSF

**Fig. 1:**
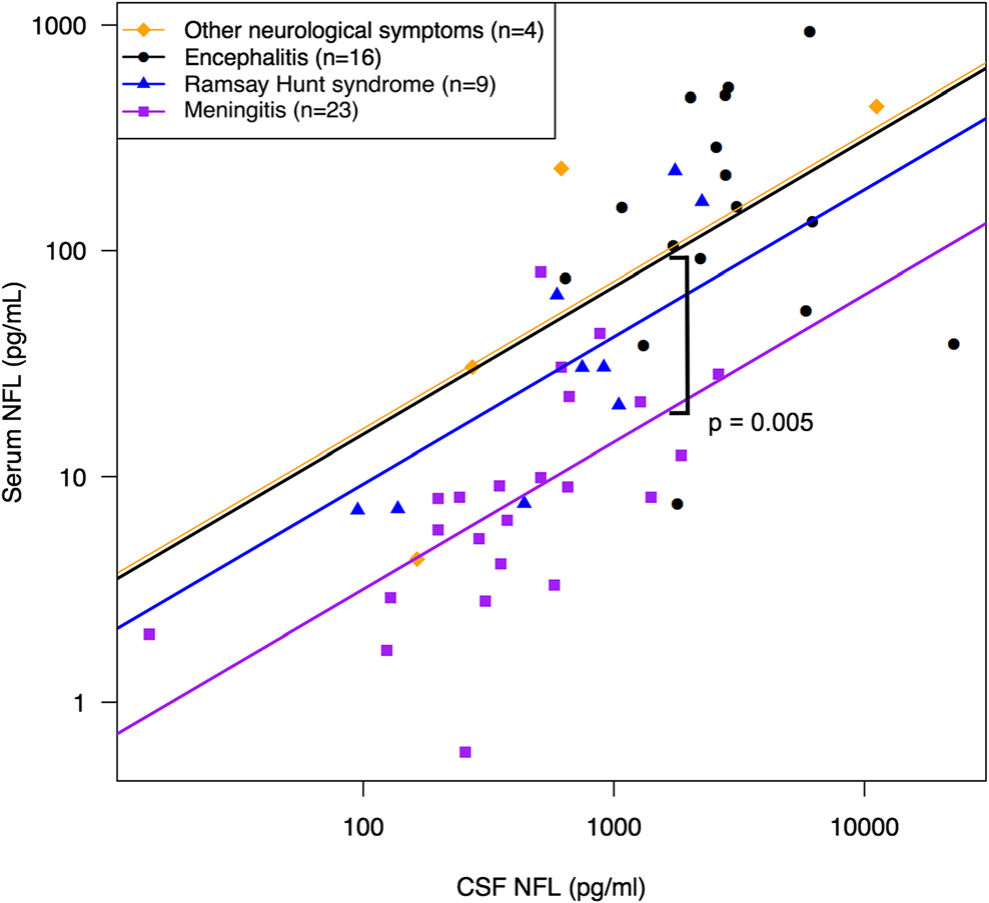
Correlation between CSF and serum NFL in 52 patients with different manifestations of VZV CNS infection (*r* = 0.72, *p* < 0.001). Subgroup analysis was made using ANCOVA. The interaction between subgroup and CSF NFL was not significant, and therefore, parallel regression lines were assumed. *CSF* cerebrospinal fluid, *NFL* neurofilament light chain

There was a strong correlation between concentrations of NFL in serum and CSF in the patients with VZV CNS infection (*r* = 0.72, *p* < 0.001) (Fig. 1). To evaluate if the correlation varied between subgroups, an interaction term between subgroup and CSF NFL was put into the model. The interaction term was not significant, and therefore, we could assume parallel regression lines. In this model, the serum/CSF NFL ratio was significantly different between the encephalitis and meningitis patients (Fig. 1). Every given CSF NFL level correlated to a mean 4.9 times higher serum NFL level in encephalitis patients than in patients with meningitis (95% confidence interval 1.4–16.6, *p* = 0.005). The patients with “other neurological symptoms” were not analysed separately as only four patients had available paired samples.

#### Serum

**Fig. 2:**
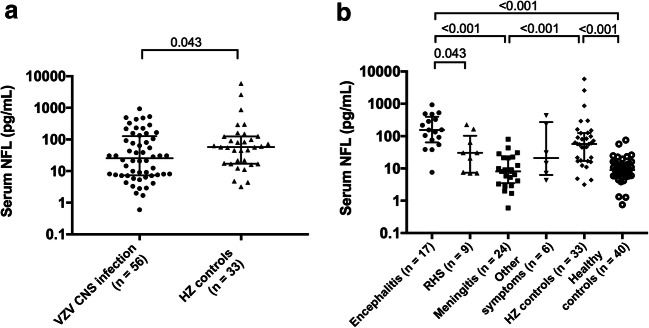
Serum NFL concentrations in the patient group with VZV CNS infection as a whole (*n* = 56) compared with HZ controls (**a**), and divided into subgroups based on diagnosis, compared with controls (**b**). Median and IQR are shown. *HZ* herpes zoster, *NFL* neurofilament light chain, *RHS* Ramsay Hunt syndrome, *VZV* varicella-zoster virus

The herpes zoster controls had higher serum NFL concentrations than the VZV CNS infection group as a whole (median 57.3 pg/mL; IQR 17.2–124.5 versus 25.5 pg/mL; 7.3–127.1, *p* = 0.043) (Fig. 2). After subdividing the patients, the encephalitis patients had significantly higher concentrations of serum NFL than those with meningitis (155.6 pg/mL; 65.1–403.8 pg/mL versus 8.1 pg/mL; 3.5–21.3 pg/mL, *p* < 0.001), Ramsay Hunt syndrome (30.4 pg/mL; 7.4–114.4 pg/mL, *p* = 0.043), and the healthy controls (9 pg/mL; 5.9–14.7 pg/mL, *p* < 0.001) (Fig. 2b). The herpes zoster controls presented significantly higher concentrations of serum NFL compared with the meningitis patients (*p* < 0.001) and healthy controls (*p* < 0.001), but not compared with the encephalitis patients (*p* = 0.22).

**Fig. 3:**
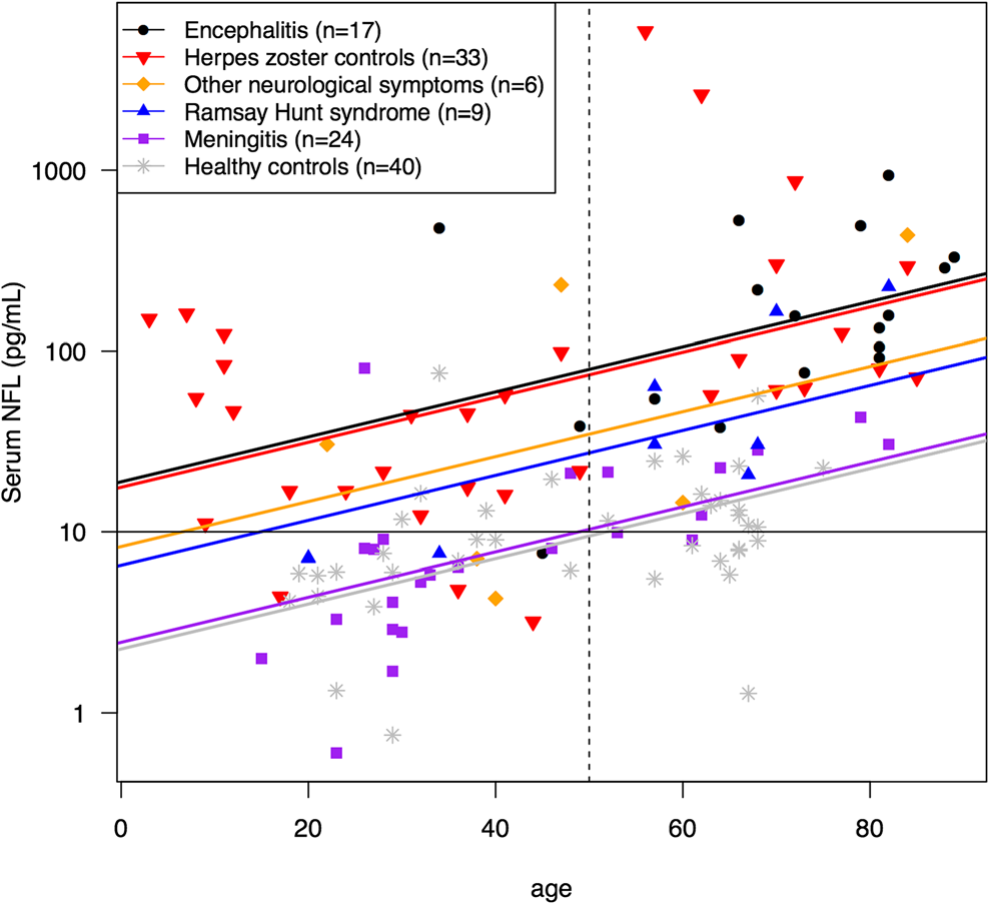
Analysis of covariance of serum NFL with age as a covariate. The dotted line represents the approximate mean age = 50 at which the group comparisons were calculated. *NFL* neurofilament light chain

No age-related cutoffs have been established for serum NFL. To adjust for age differences between the study groups in our material, we performed an analysis of covariance with age as a covariate variable and an interaction term between age and group. Since the interaction was not significant, we dropped the interaction term and assumed parallel regression lines (Fig. 3). For comparison, serum NFL concentration across all groups were adjusted to the approximate mean age = 50. The resulting adjusted serum NFL concentrations were as follows (geometric means and 95% confidence intervals are presented): encephalitis 79.4 pg/mL (CI 44.4–141.9), meningitis 10.3 pg/mL (CI 6.5–16.5), Ramsay Hunt syndrome 27.4 pg/mL (CI 12.9–58.1), “other neurological symptoms” 34.8 pg/mL (CI 13.9–87.3), herpes zoster controls 74.1 pg/mL (CI 49.7–110.4), and healthy controls 9.5 pg/mL (CI 6.6–13.6). *p* values for group comparisons are shown in Table 2.

**Table 2:** *p* values for group comparisons in the analysis of covariance of serum NFL with age as a covariate (Fig. 3). The comparison was calculated at the approximate mean age = 50

Group comparisons	*p* value
Enc vs. HZ	0.850
Enc vs. ONS	0.137
Enc vs. RHS*	0.028
Enc vs. Men*	< 0.001
Enc vs. HC*	< 0.001
HZ vs. ONS	0.138
HZ vs. RHS*	0.023
HZ vs. Men*	< 0.001
HZ vs. HC*	< 0.001
ONS vs. RHS	0.691
ONS vs. Men*	0.021
ONS vs. HC*	0.010
RHS vs. Men*	0.032
RHS vs. HC*	0.013
Men vs. HC	0.773

*Enc* encephalitis, *HC* healthy controls, *HZ* herpes zoster controls, *Men* meningitis, *NFL* neurofilament light chain, *ONS* other neurological symptoms, *RHS* Ramsay Hunt syndrome

*Statistically significant

#### CSF

**Fig. 4:**
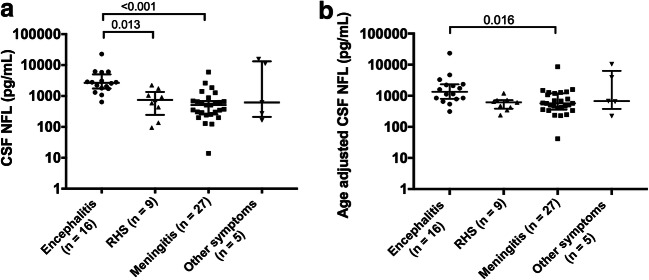
CSF NFL concentrations in 57 patients with VZV CNS infection (**a**), and adjusted to age (**b**). The approximate mean age of 50 was used for age adjustment. Median and IQR are shown. *CSF* cerebrospinal fluid, *NFL* neurofilament light chain, *RHS* Ramsay Hunt syndrome

The concentrations of NFL in CSF were significantly higher in the encephalitis patients (median 2671 pg/mL; IQR 1742–5161 pg/mL) compared with the patients with meningitis (510.9 pg/mL; 255.4–688.6 pg/mL, *p* < 0.001) and Ramsay Hunt syndrome (748 pg/mL; 288.2–1401 pg/mL, *p* = 0.013), but not compared with the patients with “other neurological symptoms” (616.7 pg/mL; 218.3–13,394 pg/mL, *p* = 0.632) (Fig. 4). An adjustment to the approximate mean age of 50 years was made across all groups (Yilmaz et al. 2017). The comparison is shown in Fig. 4b. After age adjustment, the encephalitis patients still showed significantly higher levels of CSF NFL compared with patients with meningitis (*p* = 0.016), but not to those with Ramsay Hunt syndrome (*p* = 0.062).

### Rash

**Fig. 5:**
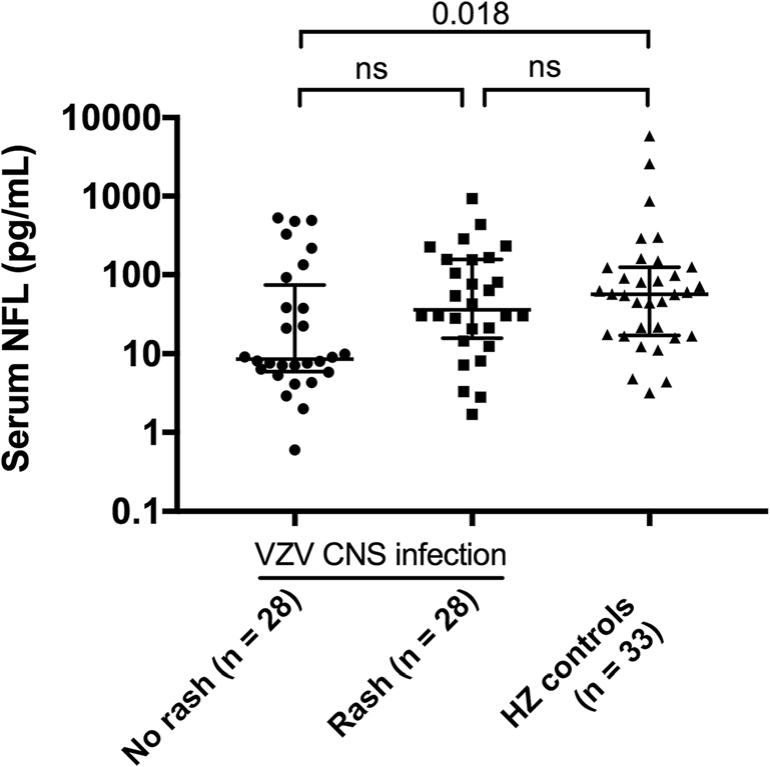
Serum NFL concentrations in the patients with VZV CNS infection, divided depending on presence of rash or not, and compared with HZ controls. Median and IQR are shown. *HZ* herpes zoster, *NFL* neurofilament light chain, *ns* not significant, *VZV* varicella-zoster virus

To investigate if the presence of a rash affects serum NFL concentration, we divided the VZV CNS infection group into patients with or without rash. Twenty-eight out of the 56 patients with VZV CNS infection (50%) that had been analysed for serum NFL had a rash. These patients had a tendency towards higher concentrations of serum NFL compared with those lacking a rash (28 out of 56 patients) although not significant (median 36.8 pg/mL; IQR 16.1–156.7 versus 8.6 pg/mL; 6–78.7, *p* = 0.139) (Fig. 5). However, the concentrations of serum NFL in the herpes zoster controls were significantly higher when comparing with the patients lacking a rash in the VZV CNS infection group (*p* = 0.018). In addition, no significant difference was shown when comparing the herpes zoster controls with the patients that had a rash in the VZV CNS infection group (57.3 pg/mL; IQR 17.2–124.5 versus 36.8 pg/mL; 16.1–156.7, *p* = 0.715). Thus, based on these results, we cannot exclude an association between rash and higher serum NFL concentrations in patients with VZV infection.

### Viral load, albumin ratio and immunosuppression

There was no significant correlation between the amount of CSF VZV DNA, i.e. viral load, and concentrations of NFL in either CSF (*r* = 0.13, *p* = 0.33) or in serum (*r* = 0.22, *p* = 0.11) in the patients with VZV CNS infection. Furthermore, multiple comparison of viral load between the different patient groups with VZV CNS infection showed significantly higher viral load only when comparing the patients with encephalitis and “other neurological symptoms” (*p* = 0.039) (see Table 1 for viral load in different groups).

**Fig. 6:**
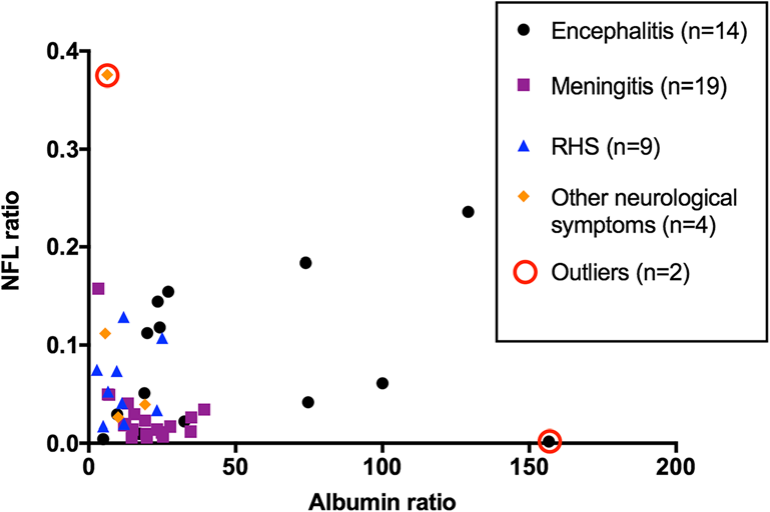
Correlation between NFL ratio (serum NFL/CSF NFL) and albumin ratio (*r* = 0.42, *p* = 0.004). Two outliers were excluded prior to correlation analysis. Subgroup analysis showed that there was a significant negative correlation in the meningitis patients (*r* = − 0.48, *p* = 0.04). *NFL* neurofilament light chain

To evaluate if the serum NFL concentrations were depending on the degree of blood-brain barrier damage in the patients with VZV CNS infections, the serum/CSF NFL ratios were analysed for correlation with the albumin ratios. No correlation was found when including all values (*r* = 0.11, *p* = 0.45). Yet, a scatter plot indicated a possible influence of two outliers (Fig. 6), and after exclusion of the outliers, a positive correlation was shown (*r* = 0.42, *p* = 0.004). However, further subgroup analysis showed that there was a significant negative correlation in the meningitis patients (*r* = − 0.48, *p* = 0.04). The CSF NFL concentrations and albumin ratios correlated (*r* = 0.48, *p* < 0.001), whereas a less strong but significant correlation was found between the serum NFL concentrations and albumin ratios (*r* = 0.29, *p* = 0.04).

The influence of immunosuppression on serum NFL concentrations was also evaluated. Within the VZV CNS infection group, the immunocompromised patients presented higher concentrations of serum NFL (median 156.2 pg/mL; IQR 41.8–515.2 pg/mL) versus the immunocompetent patients (20.9 pg/mL; 7.1–84.9 pg/mL) (*p* = 0.0039). In the herpes zoster control group, the concentrations of serum NFL were not significantly different between immunocompromised and immunocompetent patients (60.8 pg/mL; 44.8–155.2 pg/mL versus 35.2 pg/mL; 16.2–88.7 pg/mL, *p* = 0.319).

## Discussion

This study is to our knowledge the first to examine serum NFL concentrations in patients with VZV CNS infection. Our results indicate that analysis of serum NFL may be used instead of analysis of CSF for estimation of neuronal injury in patients with VZV CNS infection and possibly also in other viral CNS infections. The finding of a strong correlation between serum and CSF NFL is in agreement with recent studies including patients with CNS injury in HIV infection (Gisslen et al. 2016), multiple sclerosis (Disanto et al. 2017; Piehl et al. 2018) and traumatic brain injury (Shahim et al. 2016). In addition, serum NFL seems to correlate well with CSF NFL within each clinical subgroup, but the ratio between serum and CSF NFL varied depending on the clinical manifestation (Fig. 1). The encephalitis patients showed higher levels of serum/CSF NFL ratio compared with the meningitis patients, and therefore, comparison of serum NFL levels between subgroups should be made with caution. Moreover, this difference raised the question whether the degree of blood-brain barrier damage influenced the serum/CSF NFL ratio.

Both CSF and serum NFL concentrations correlated with increasing blood-brain barrier damage measured by the CSF/serum albumin ratio, as previously shown in studies of dementias and in CNS injury in HIV (Gisslen et al. 2016; Skillback et al. 2017). One may assume that in CNS infections, increasing blood-brain barrier damage would cause increased leakage of NFL from CSF to serum, leading to an increasing serum/CSF NFL ratio in conjunction with increasing albumin ratio. However, our results were contradictory, as a positive correlation was found when including all patients except outliers, but a negative correlation was seen in the subgroup of meningitis. A recent study by Kalm et al. (Kalm et al. 2017) found no influence of blood-brain barrier damage on serum NFL levels. Another theory is that the degree of blood-brain barrier damage is a general marker of disease severity and therefore correlates with the concentrations of NFL in both serum and CSF but not with serum/CSF NFL ratio. In severe CNS disease, there is probably an increased risk of both blood-brain barrier damage and considerable injury of neurons (Anesten et al. 2016).

The encephalitis patients had the highest serum NFL concentrations when compared with the patients with meningitis and Ramsay Hunt syndrome. Encephalitis is considered the most severe manifestation of VZV CNS infection. Indeed, higher concentrations of serum NFL have been correlated to increased disease activity in several neurological diseases such as multiple sclerosis (Disanto et al. 2017), amyotrophic lateral sclerosis (Lu et al. 2015) and CNS injury in HIV infection (Gisslen et al. 2016). However, unexpectedly, the herpes zoster control patients without CNS manifestations had very high concentrations of serum NFL, comparable with the levels seen in the encephalitis patients. This leads to the assumption that peripheral nerve damage increases serum NFL levels in patients with rash due to VZV. Peripheral nerves produce NFL (Trojanowski et al. 1986) and can be damaged due to herpes zoster (Schmidbauer et al. 1992). Furthermore, serum NFL concentrations are increased in inherited peripheral neuropathies (Sandelius et al. 2018) and Guillain-Barré syndrome (Gaiottino et al. 2013). Interestingly, the latter study also showed increased CSF NFL levels, supposedly related to peripheral nerve degradation. In older autopsy studies of patients with herpes zoster (Schmidbauer et al. 1992), it seems like the neuronal damage also include nerve roots situated in the spinal canal, which may yield increased CSF NFL concentrations also in herpes zoster patients without clinical symptoms of CNS disease. Additional studies that include CSF samples from herpes zoster patients would be of interest to further elucidate this matter.

In conjunction with high levels of serum NFL, the patients with encephalitis also demonstrated higher concentrations of CSF NFL compared with the patients with meningitis and Ramsay Hunt syndrome. After age adjustment, this difference remained in comparison with that of the meningitis patients. Encephalitis patients have been reported to have high concentrations of CSF NFL also in a previous study (Grahn et al. 2013). In that study, no significant differences in CSF NFL concentrations were found between encephalitis and meningitis patients, but the patient groups were smaller than in the present study. In encephalitis, the infection reaches into the brain, with a large number of neurons, whereas in meningitis, it is mainly the meninges that are involved, with far less neurons. Therefore, higher concentrations of NFL in CSF and serum in patients with encephalitis seem logic, as they reflect the number of neurons that are affected. In addition, it is reasonable to assume different pathogenesis between these two CNS manifestations, as supported by recent findings of different chemokine response (Lind et al. 2019) and higher viral diversity in encephalitis versus meningitis caused by VZV (Depledge et al. 2018).

The influence of immunosuppression on levels of NFL is unclear. In our material, the immunocompromised patients in the VZV CNS infection group showed higher levels of serum NFL than immunocompetent patients. In contrast, this was not shown in the herpes zoster control group. As the inflammatory response differ between the CNS and the peripheral nervous system in patients with CNS infections caused by herpes simplex virus and VZV (Lind et al. 2019; Lind et al. 2017), we can only speculate that immunosuppression may affect the inflammatory response in different ways in the central and peripheral nervous systems in these patients.

Finally, as the serum NFL assay means much less harm for the patient by avoiding a lumbar puncture, this assay can be a convenient tool for assessing the prognosis and for monitoring of disease in patients with VZV CNS infection and probably also in other viral CNS infections. However, longitudinal studies are warranted for evaluation of this purpose.

In conclusion, our findings demonstrate a strong correlation between serum and CSF NFL, suggesting that analysis of serum NFL could be used instead of CSF NFL for evaluation of neuronal injury in VZV CNS infection. However, high levels of serum NFL were also seen in patients with herpes zoster without signs of CNS involvement, indicating an influence of peripheral nerve damage on serum NFL, which could complicate the interpretation. In addition, we found significantly higher concentrations of NFL in both serum and CSF in patients with encephalitis compared with that of meningitis which strengthens the argument of using the serum NFL assay in prognostic purpose in VZV CNS infections.
